# 1158. Immunogenicity of COVID-19 vaccine four-dose series in healthy community dwelling adults 50 years and above: interim analysis from the ‘PRospEctiVe EvaluatioN of immuniTy after COVID-19 vaccines’ (PREVENT-COVID) study

**DOI:** 10.1093/ofid/ofad500.998

**Published:** 2023-11-27

**Authors:** Gabrielle N Gaultier, Brynn McMillan, Mandy Lo, Bing Cai, Jean Zheng, Hennady Shulha, Karen Simmons, Ana Citlali Marquez, Sofia R Bartlett, Inna Sekirov, James Zlosnik, Muhammad Morshed, Danuta Skowronski, Agatha N Jassem, Manish Sadarangani

**Affiliations:** University of British Columbia, Vancouver, BC, Canada; University of British Columbia, Vancouver, BC, Canada; University of British Columbia, Vancouver, BC, Canada; University of British Columbia, Vancouver, BC, Canada; University of British Columbia, Department of Microbiology and Immunology, Richmond, British Columbia, Canada; Vaccine Evaluation Center, BC Children's Hospital Research Institute, The University of British Columbia, Vancouver, British Columbia, Canada; University of British Columbia, Vancouver, BC, Canada; BC Centre for Disease Control, Vancouver, British Columbia, Canada; BC Centre for Disease Control, Vancouver, British Columbia, Canada; University of British Columbia, Vancouver, BC, Canada; BC Centre for Disease Control/ University of British Columbia Department of Pathology and Laboratory Medicine, Vancouver, British Columbia, Canada; University of British Columbia, Vancouver, BC, Canada; BC Centre for Disease Control, Vancouver, British Columbia, Canada; University of British Columbia, Vancouver, BC, Canada; University of British Columbia, Vancouver, BC, Canada

## Abstract

**Background:**

Adults aged > 50 years are at increased risk for severe coronavirus disease 2019 (COVID-19). This study evaluated immunogenicity of COVID-19 vaccines in healthy adults aged > 50 years through quantification of antigen specific antibody concentration and function post-vaccination with two, three and four COVID-19 vaccine doses.

**Methods:**

Eighty-four immunocompetent, community-dwelling adults 50 to 83 years old (median age 61 years) were enrolled. We measured index virus spike protein (S) specific antibody responses to mRNA (mRNA-1273 or BNT162b2) and/or ChAdOx1-S COVID-19 vaccines. Participants were separated into three groups: (1) mRNA/mRNA/mRNA/mRNA; (2) ChAdOx1-S/mRNA/mRNA; (3) ChAdOx1-S/ChAdOx1-S/mRNA. Responses were quantified via: anti-S IgG geometric mean concentrations (GMCs) (binding antibody units [BAU]/mL), total relative IgG avidity index (TRAI) (avidity units, AU) collected up to one, four, and seven-months after each dose. One-way ANOVA, Tukey-Kramer post-hoc compared groups and Welch’s t-test compared timepoints.

**Results:**

At one month post-dose two, mRNA/mRNA (1137 BAU/mL, p = 0.0003) and ChAdOx1-S/mRNA (1388, p < 0.0001) had higher anti-S IgG GMCs compared with ChAdOx1-S/ChadOx1-S (195 BAU/mL). However, TRAI was similar amongst the three groups (all p > 0.05); mRNA/mRNA (70 AU), ChAdOx1-S/mRNA (66 AU) and ChAdOx1-S/ChAdOx1-S (58 AU). S-IgG GMCs at one month post-dose three were higher for mRNA/mRNA/mRNA than ChAdOx1-S/ChAdOx1-S/mRNA (1316 vs. 569 BAU/mL p = 0.0162). Similar to post-dose two, post-dose three there were no significant differences in TRAI between groups (all p > 0.05); mRNA/mRNA/mRNA (95 AU), ChAdOx1-S/mRNA/mRNA (98 AU), ChAdOx1-S/ChAdOx1-S/mRNA (101 AU). For mRNA/mRNA/mRNA participants, a fourth mRNA vaccine dose increased S-IgG GMCs at one-month post-dose four compared with one month post-dose two (2825 vs. 1137 BAU/mL, p = 0.0126) and maintained TRAI between timepoints (71 vs. 70 AU, p = 0.9877).

**Conclusion:**

mRNA vaccines are more immunogenic compared to ChAdOx1-S with regards to S-specific antibody concentration. mRNA boosters maintained antibody avidity. Together, TRAI and anti-S IgG GMCs should be further evaluated when recommending additional booster doses and considered when determining protection against COVID-19.

**Disclosures:**

**Sofia R. Bartlett, PhD**, Abbvie: Advisor/Consultant|Abbvie: Grant/Research Support|Cepheid: Advisor/Consultant|Gilead: Advisor/Consultant|Gilead: Grant/Research Support **Manish Sadarangani, BM BCh, FRCPC, DPhil**, GlaxoSmithKline: Grant/Research Support|Merck: Grant/Research Support|Moderna: Grant/Research Support|Pfizer: Grant/Research Support|Sanofi Pasteur: Grant/Research Support|Seqirus: Grant/Research Support|Symvivo: Grant/Research Support|VBI Vaccines: Grant/Research Support

